# A Case of Preternatural Growth of the Gums

**Published:** 1850-07

**Authors:** J. Taft


For the Dental Register.

### A CASE OF PRETERNATURAL GROWTH OF THE GUMS.

Among the numerous diseases to which the gums are subject, the preternatural growth, combined with an inflammatory sponginess, is an important one. It is an important one, because serious and sometimes fatal consequences are involved in it; its progress is oftentimes very rapid; anything less than thorough treatment will prove unavailing, so far as a permanent cure is concerned. It is always a matter of great interest to study and effectually treat those afflicting maladies and ills to which human flesh is heir.

This disease, its cause, effect, and treatment, may be illustrated by the following case. "This case is not given because the disease is seldom met with; nor is it presumed here that the disease is but rarely thoroughly treated; but it is given because each feature is a prominent one—every point a strong one; this refers to the disease and not to the treatment—and because of the advanced stage to which it had been permitted to extend, as it must very soon have proved fatal, had it not been arrested.

I was called upon to visit the patient about the 15th of May, 1849—aged about twenty-five years. The temperament was a combination of the sanguine and the lymphatic, the former predominating.

When I first saw her she was in a very enfeebled condition, confined to her bed the greater part of the time—the prostration had been coming on rapidly for about four weeks. It was about two months previous to the examination, that the patient observed anything unnatural with the gums; at which time a slight burning sensation was experienced, which continued to increase until pain almost intolerable was the result. The gums were not very sore to the touch, but pressure augmented the thrill of pain through the parts. The condition of the teeth is worthy of notice. There were but few sound teeth in the mouth, and the roots of all the other teeth were remaining in the mouth, some with the crowns but partially decayed away, and others decayed to or below the necks; they were, many of them, quite loose, so that they were easily removed with a curved excavator; they were only retained in their position by the enlarged condition of the gums over them; others still had some attachment to the sockets. The teeth had decayed and broken away without causing much pain, so far as an aching condition was concerned; they were all to some extent enveloped in pus. The gums at the margin or free border, was a dark purple, or rather black; receding from the margin, they became of slightly brighter color, but they were of a dark purple to the attachment of the soft parts externally, the whole mucous membrane of the mouth was involved to some extent. The volume of the gums was increased to three or four times the normal size, so that it was impossible for the patient to bring the jaws to their natural position; so much were they enlarged, that they covered the crowns of those teeth that were not decayed, the points of one or two only protruding. At some points the gums would bleed at the slightest puncture, though the most dark and pendant parts would bleed but sluggishly; the margin was quite ragged and uneven.

A general prostration of the system had been developed along with the progress of the disease. This prostration was marked by a failure of nervous energy, dizziness, headache loss of appetite, and finally by an indescribable nausea. Such had been the condition of the mouth for some time, that food requiring mastication could not be taken, from the fact that mastication could not be performed. There was, however, no appetite for food. The odor from the gums was extremely offensive—the nausea of the patient might in some degree have resulted from the offensive odor.

It is not necessary to say much about the causes that operate to produce this condition. Doubtless it arises, in all cases, in the out-start from local causus; though a scorbutic or scrofulous tendency would accelerate its progress, and that too, in an increased ratio as the disease progressed; so that in an advanced stage of the disease, a scorbutic diathesis, if action might exercise the controling influence. In the case under consideration, the cause appeared to have been, in the outset, entirely local, for there appeared to be a perfect freedom from anything like a cachectic diathesis.

The treatment in this case was just such as I suppose would have been suggested by every one acquainted with the disease. The first impression upon the mind is prompt and thorough treatment. That the constitutional symptoms might be relieved somewhat before the operation, Comp. Ext. Colocymth was prescribed for two or three days, which modified the character of the symptoms very much, after which the operation upon the mouth was commenced. I anticipated excessive hemorrhage, which, however, was not realized to any great extent. The operation was commenced by removing from the upper jaw all the decayed teeth and roots; and then with the knife placed some half an inch or more above the margin of the gum, and about at that point which the free border occupies in health; it was passed through and round parallel with the margin, until all that portion below the line of the knife was separated; the operation was performed in the same manner upon the lower jaw. After the operation, the mouth was frequently washed with a compound tincture of myrrh, prepared from the simple tinctures of myrrh, opium and capsicum, at first very much diluted; this was used for two days; when the mouth was again examined, and such other portions of gum removed as was thought necessary; the above preparation with some modification was continued. About four days after the operation, I began the application of nitrate of silver, this was applied repeatedly. The result of this treatment was, that in a few days the constitutional derangements all disappeared; the patient, quite relieved from the pain and suffering she had endured, and at the expiration of one month she had entirely recovered from that disease which had so nearly prostrated her. And now, which is fourteen months after the operation, she enjoys good health, and a buoyancy of spirit that is not often met with, and the gums and remaining teeth are in a perfectly healthy condition. J. TAFT.

